# Targeted delivery of 5-fluorouracil-1-acetic acid (5-FA) to cancer cells overexpressing epithelial growth factor receptor (EGFR) using virus-like nanoparticles

**DOI:** 10.1038/s41598-020-73967-4

**Published:** 2020-10-08

**Authors:** Bee Koon Gan, Kamal Rullah, Chean Yeah Yong, Kok Lian Ho, Abdul Rahman Omar, Noorjahan Banu Alitheen, Wen Siang Tan

**Affiliations:** 1 Institute of Bioscience, Universiti Putra Malaysia, 43400 UPM Serdang, Selangor Malaysia; 2 Department of Pharmaceutical Chemistry, Kulliyyah of Pharmacy (KOP), International Islamic University Malaysia (IIUM), 25200 Kuantan, Pahang Malaysia; 3 Department of Pathology, Faculty of Medicine and Health Sciences, Universiti Putra Malaysia, 43400 UPM Serdang, Selangor Malaysia; 4 Department of Veterinary Pathology and Microbiology, Faculty of Veterinary Medicine, Universiti Putra Malaysia, 43400 UPM Serdang, Selangor Malaysia; 5 Department of Cell and Molecular Biology, Faculty of Biotechnology and Biomolecular Sciences, Universiti Putra Malaysia, 43400 UPM Serdang, Selangor Malaysia; 6 Department of Microbiology, Faculty of Biotechnology and Biomolecular Sciences, Universiti Putra Malaysia, 43400 UPM Serdang, Selangor Malaysia

**Keywords:** Nanobiotechnology, Peptide delivery, Nanobiotechnology, Nanomedicine

## Abstract

Chemotherapy is widely used in cancer treatments. However, non-specific distribution of chemotherapeutic agents to healthy tissues and normal cells in the human body always leads to adverse side effects and disappointing therapeutic outcomes. Therefore, the main aim of this study was to develop a targeted drug delivery system based on the hepatitis B virus-like nanoparticle (VLNP) for specific delivery of 5-fluorouracil-1-acetic acid (5-FA) to cancer cells expressing epithelial growth factor receptor (EGFR). 5-FA was synthesized from 5-fluorouracil (5-FU), and it was found to be less toxic than the latter in cancer cells expressing different levels of EGFR. The cytotoxicity of 5-FA increased significantly after being conjugated on the VLNP. A cell penetrating peptide (CPP) of EGFR was displayed on the VLNP via the nanoglue concept, for targeted delivery of 5-FA to A431, HT29 and HeLa cells. The results showed that the VLNP displaying the CPP and harboring 5-FA internalized the cancer cells and killed them in an EGFR-dependent manner. This study demonstrated that the VLNP can be used to deliver chemically modified 5-FU derivatives to cancer cells overexpressing EGFR, expanding the applications of the VLNP in targeted delivery of chemotherapeutic agents to cancer cells overexpressing this transmembrane receptor.

## Introduction

5-fluorouracil (5-FU), an analog of pyrimidine, is one of the most effective antineoplastic agents, which shows remarkably enhanced inhibitory effects against a wide range of solid tumors^1–3^. 5-FU restrains the proliferation of cancer cells by inhibiting thymidylate synthase, and incorporating its metabolites into RNA and DNA^4^. Nonetheless, the inappropriate oral absorption and reduced bioavailability of 5-FU frequently lead to disappointing clinical therapeutic outcomes^5–7^. In addition, 5-FU also causes a variety of unfavorable effects including dermatitis, mucositis, myelosuppression, nausea, vomiting, diarrhea and gastrointestinal problems. These unfavorable effects are mainly due to the lack of specificity towards cancer cells^8–10^. Therefore, developing proper strategies to achieve targeted and efficient uptake of 5-FU into cancer cells is of critical importance to augment the effectiveness, and lower the undesirable effects of 5-FU.

In order to achieve targeted drug delivery, cell penetrating peptides (CPPs) that penetrate cell membranes via specific interactions with cell surface receptors have become increasingly popular for the design of an ideal drug delivery system^11–13^. Epithelial growth factor receptor (EGFR), a transmembrane receptor, which is expressed abundantly in a number of tumor cells, and highly related to angiogenesis invasion and metastasis, has been studied intensively as a potential target for cancer therapeutics^14^. CPPs that interact specifically with EGFR can serve as targeting ligands in the treatments of patients with EGFR-positive malignancies, for targeted delivery of therapeutic agents such as anticancer drugs, siRNA, and small molecules into cells overexpressing EGFR. In our previous study, a CPP with the amino acid sequence NRPDSAQFWLHH that interacts specifically with EGFR was isolated from a phage displayed peptide library via biopanning against A431 human squamous carcinoma cell^13^. Further characterization revealed that it entered the A431 cells through clathrin-dependent endocytosis^13^.

Over the past decade, delivery systems using nanoparticles, such as cationic liposomes, polymers, carbon nanotubes and virus-like nanoparticles (VLNPs) have been developed with the aim to improve therapeutic efficacies of anticancer drugs, while minimizing their undesirable side effects^15–21^. Among these nanoparticles, VLNPs demonstrate the potential for the delivery of a broad spectrum of chemotherapeutics, owing to their favorable characteristics, including (1) biocompatibility and biodegradability^22^, (2) homogeneity with specific compositions and molecular structures^23^, (3) self-assembling into nanoparticles with relatively large cavity^24^, (4) their structures, properties and functions can be tailored easily by protein engineering and recombinant DNA techniques^25^, and (5) multivalency for chemical functionalizations or genetic modifications^26,27^.

Hepatitis B VLNP comprising 180 or 240 subunits of the viral core antigen (HBcAg) has been studied intensively in the development of multicomponent vaccines and drug delivery systems^28–30^. A truncated HBcAg (tHBcAg), a mutant without the C-terminal arginine rich domain, also self-assembles into icosahedral VLNP^31–33^. The tHBcAg VLNP is highly stable and robust, and it possesses a large surface area containing a variety of amino acid residues with different functional groups^28,33^. Hence, various targeting ligands can be displayed easily on the surface of tHBcAg VLNPs to attain targeted drug delivery for cancer therapeutics^11,13,30,34,35^.

**Scheme 1:**
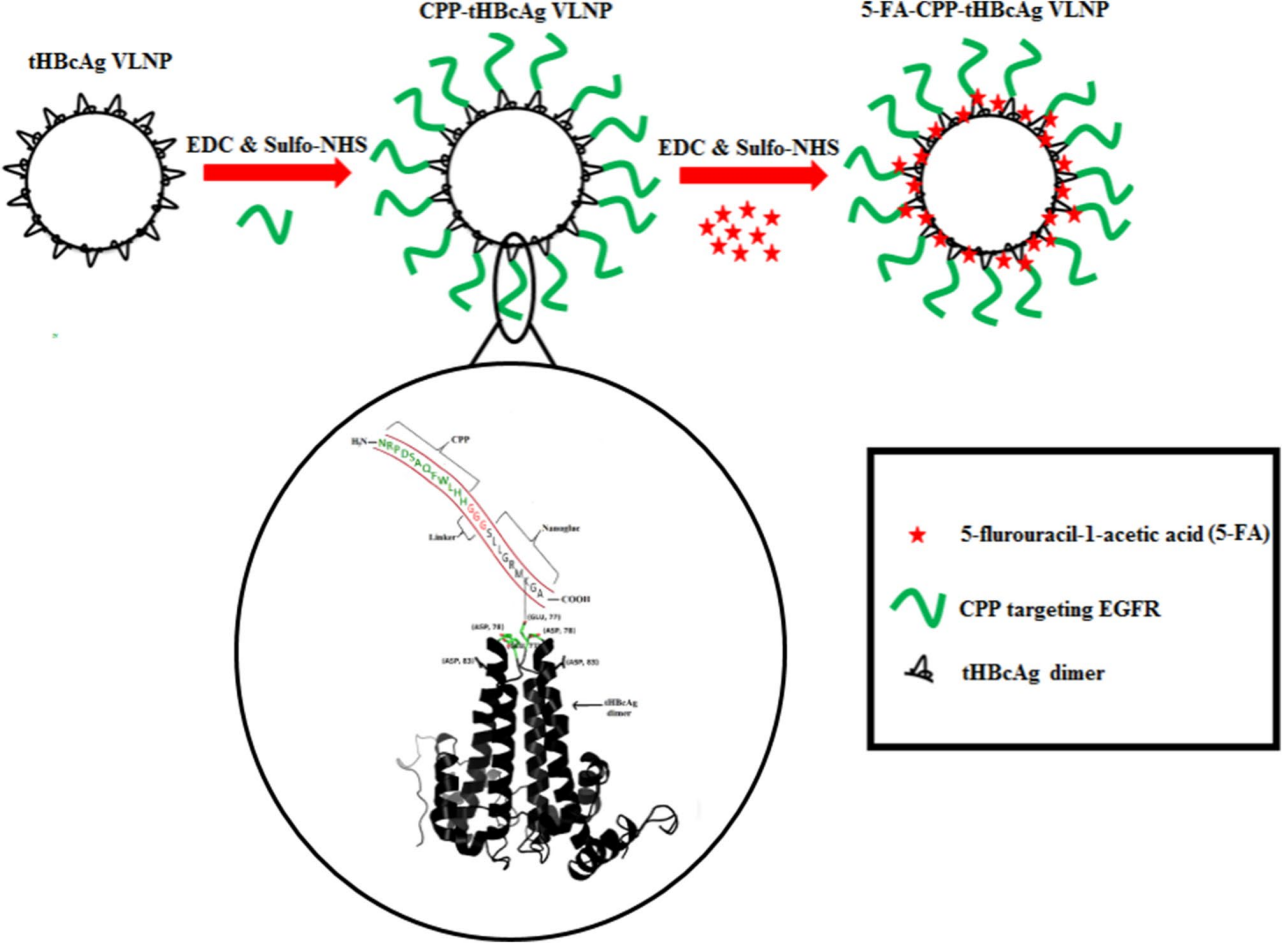
Schematic representation of dual conjugated tHBcAg VLNP for targeted drug delivery. tHBcAg VLNP exhibits a number of Glu, Asp and Lys residues accessible for the conjugation of drugs and cancer targeting moieties using EDC and sulfo-NHS. Carboxyl groups of Glu and Asp located at the spike of the dimer of tHBcAg VLNP were covalently linked to the primary amines of Lys residue on the nanoglue (SLLGRMKGA) co-synthesized with the CPP (NRPDSAQFWLHH) targeting EGFR. The two sequences are separated by a linker (GGG). The CPP-tHBcAg VLNP was then conjugated with 5-FA at Lys residues exposed on the surface of tHBcAg VLNP. The three-dimensional structure of tHBcAg dimer was prepared using PyMOL^68^ (The PyMOL Molecular Graphics System; https://www.pymol.org).

In this study, a derivative of 5-FU, 5-fluorouracil-1-acetic acid (5-FA) that is less toxic than the former was synthesized. The 5-FA and CPP (NRPDSAQFWLHH) were conjugated on the surface of tHBcAg VLNP using 1-ethyl-3-(3-dimethylaminopropyl) carbodiimide hydrochloride (EDC) and sulfo-N-hydroxysuccinimide (Sulfo-NHS) (Scheme 1). The cytotoxicity of the newly developed tHBcAg VLNP harboring 5-FA and CPP was then compared with that of free 5-FA and 5-FU in cancer cells (A431, HT29 and HeLa cells) expressing different levels of EGFR^36^, in which the conjugate demonstrated enhanced cytotoxicity towards the cells expressing higher level of EGFR, particularly the A431 cells.

## Results

### Analysis of EGFR expression via immunofluorescence microscopy, and selective internalization property of CPP (NRPDSAQFWLHH) in cell lines expressing different levels of EGFR

**Figure 1:**
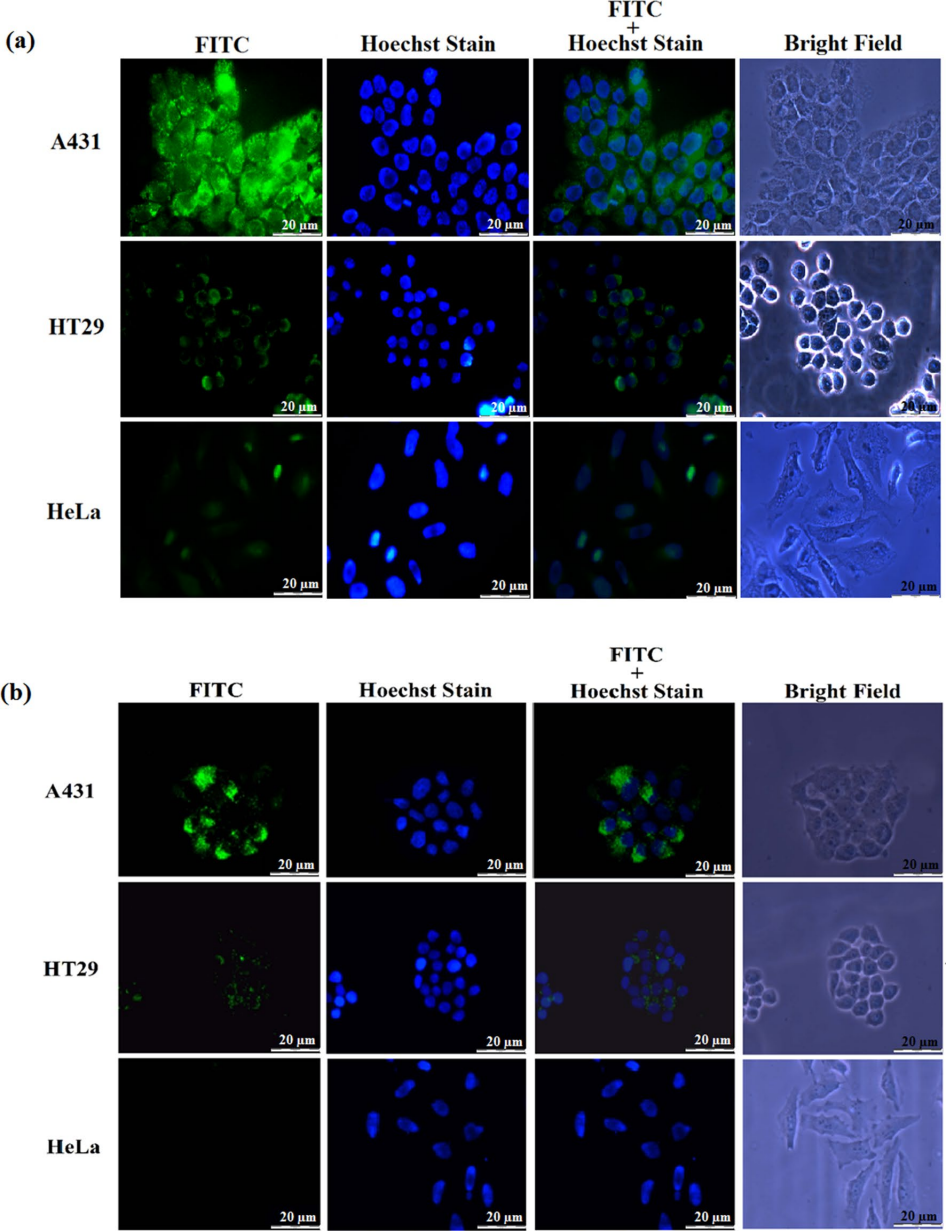
Analysis of EGFR expression via immunofluorescence microscopy, and internalization of CPP-FITC into A431, HT29 and HeLa cells expressing different levels of EGFR. (**a**) A431, HT 29 and HeLa cells were incubated with the rabbit anti-EGFR monoclonal antibody for 2 h at RT, followed by incubation with the goat anti-rabbit IgG conjugated to FITC for 1 h at RT to detect the expression of EGFR in the three cell lines. A431 cells exhibited the highest fluorescent intensity followed by HT29 cells, and HeLa cells showed the lowest fluorescent intensity. (**b**) Peptide NRPDSAQFWLHH conjugated with FITC (CPP-FITC; 0.25 mM) was added onto A431, HT29 and HeLa cells, incubated for 16 h at 37 °C, and observed under a fluorescence microscope. A431 cells that express the highest level of EGFR exhibited an intense fluorescence as compared to HT29 cells, which have intermediate amount of EGFR. The fluorescent signal was negligible in HeLa cells, which have the lowest number of EGFR/cell.

To detect the expression of EGFR in A431, HT29 and HeLa cells, the cell lines were incubated with the rabbit anti-EGFR monoclonal antibody, Cetuximab (C225), followed by incubation with the goat anti-rabbit IgG conjugated to FITC. Figure 1a shows that A431 cells exhibited the highest green fluorescent intensity, indicating the highest expression level of EGFR in A431 cells. The fluorescent intensity in HT29 cells is lower as compared to A431 cells, while HeLa cells showed the lowest fluorescent intensity. This indicates that the expression level of EGFR in HT29 cells is lower than that in A431 cells, while HeLa cells expressed the lowest level of EGFR among the three cell lines. In order to elucidate the targeting property of CPP with the sequence NRPDSAQFWLHH towards EGFR, the FITC-conjugated CPP was incubated simultaneously with three types of cell lines expressing different levels of EGFR: A431, HT29 and HeLa. As shown in Fig. 1b, A431 cells exhibited an intense green fluorescence as compared with HT29 cells, while HeLa cells showed the lowest intensity of green fluorescence. These results indicated that the CPP internalized efficiently into A431 cells expressing the highest level of EGFR, followed by HT29 cells, which have intermediate amount of EGFR. The uptake of the CPP reduced drastically in HeLa cells, which have the lowest expression level of EGFR among these cell lines. The results demonstrated that the CPP is targeted towards EGFR for cell internalization.

### Conjugation of CPP to tHBcAg VLNP via the nanoglue, and cellular uptake of the conjugated nanoparticle into A431, HT29 and HeLa cell lines

**Figure 2:**
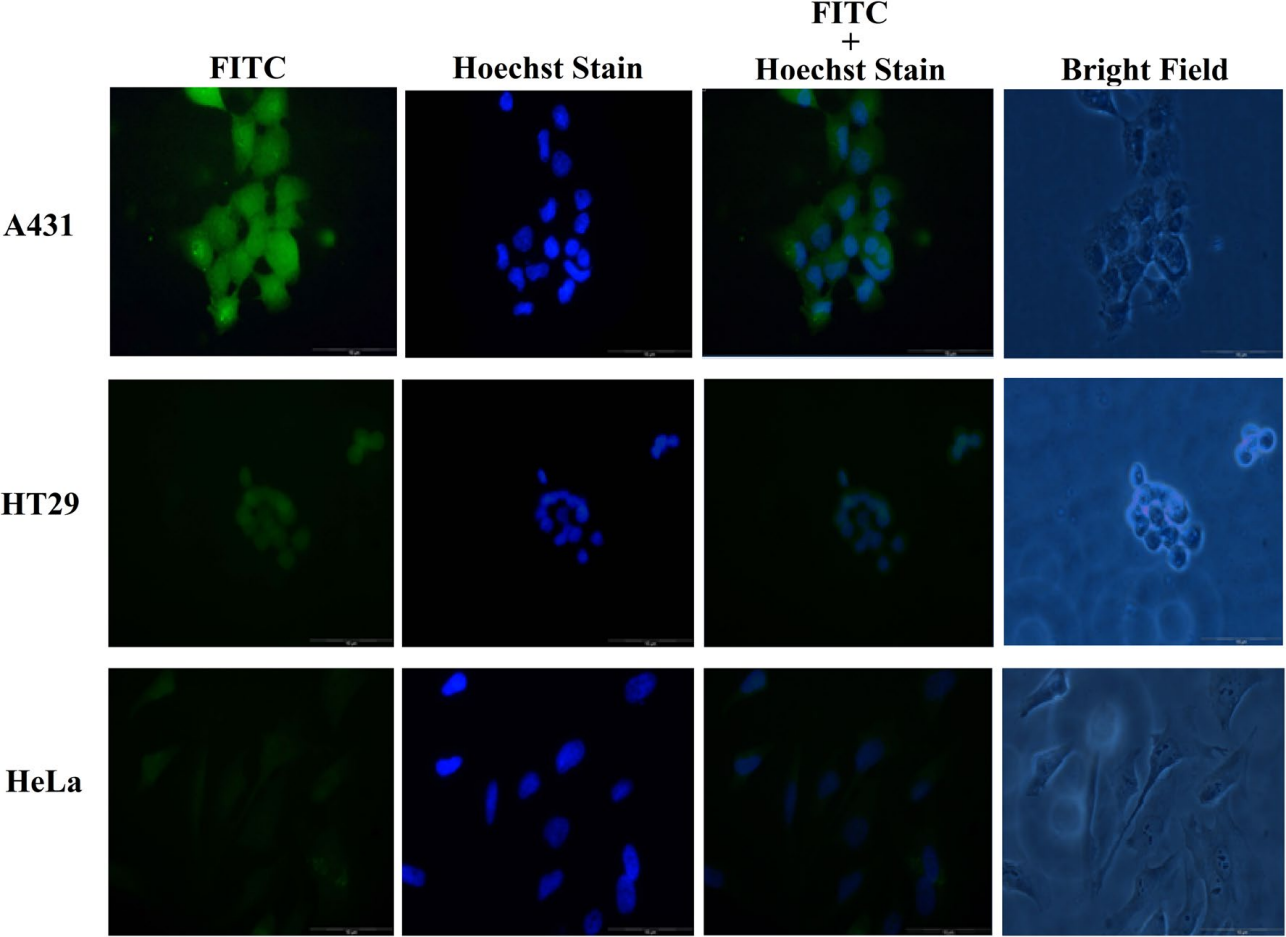
Delivery of CPP-tHBcAg VLNP into A431, HT29 and HeLa cells. CPP was conjugated covalently to tHBcAg VLNP using EDC and sulfo-NHS. The internalization of CPP-tHBcAg VLNP was identified using the mouse anti-HBcAg monoclonal antibody and the FITC-conjugated goat anti-mouse antibody. A431 cells incubated with the CPP-tHBcAg VLNP exhibited strong green fluorescent dots as compared to HT29 and HeLa cells, in accordance with the EGFR expression level of the cell lines.

The CPP (NRPDSAQFWLHH) was synthesized together with the nanoglue (SLLGRMKGA), and separated by a linker (GGG) between these two sequences. The resulting peptide, NRPDSAQFWLHHGGGSLLGRMKGA, was covalently cross-linked to the carboxyl group at the spikes of tHBcAg VLNP using Sulfo-NHS and EDC. The tHBcAg monomer shifted approximately 1 kDa on an SDS–polyacrylamide gel, demonstrating that the peptide was successfully cross-linked to the monomer (Supplementary Fig. S1). The tHBcAg VLNP displaying the CPP (namely, CPP-tHBcAg VLNP) was then incubated with A431, HT29 and HeLa cells. The internalization efficiency of the CPP-tHBcAg VLNP into the three cell lines was examined with immunofluorescence microscopy, using the mouse anti-HBcAg monoclonal antibody as the primary antibody and the FITC-conjugated goat anti-mouse antibody as the secondary antibody. Figure 2 shows that A431 cells gave rise to the highest green fluorescence signal as compared with HT29 cells, while HeLa cells had the lowest signal. This indicates that the internalization of CPP-tHBcAg VLNP into these cells is in EGFR-dependent manner. The result also demonstrated that the CPP was capable to deliver tHBcAg VLNP into cells expressing EGFR.

### Synthesis and characterization of 5-FA

**Scheme 2:**
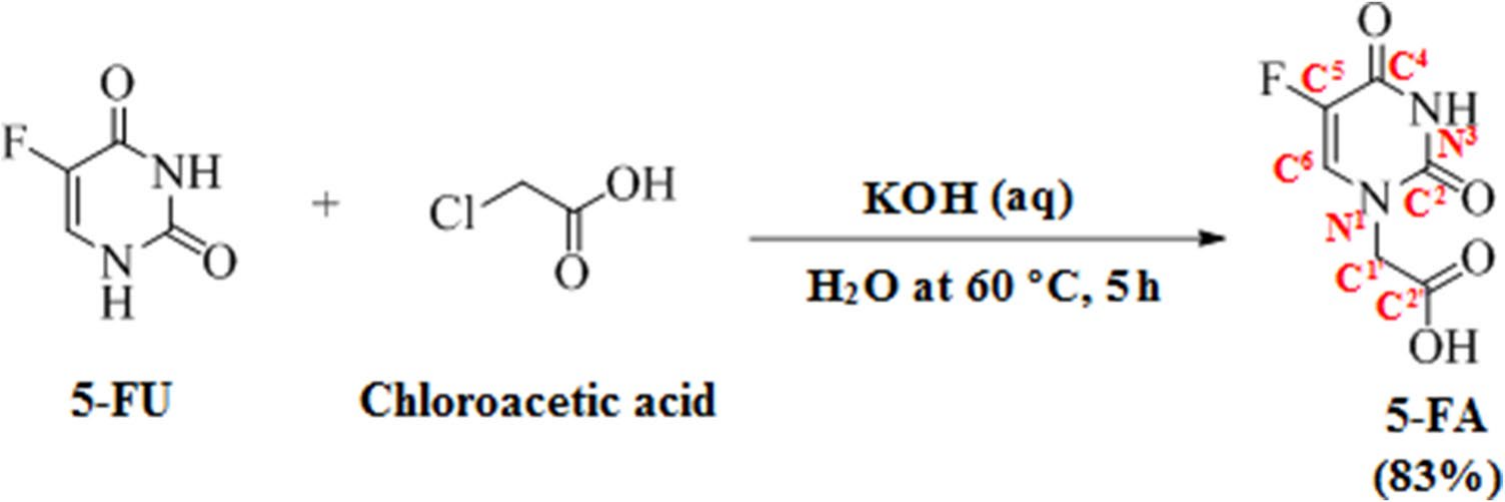
The conversion of 5-FU to 5-FA. 5-FU was reacted with α-chloroacetic acid in the presence of potassium hydroxide (KOH) and water at 60 °C for 5 h to yield 83% of 5-FA. The chemical structures were drawn using ChemDraw 15.0 (https://www.perkinelmer.com/uk/category/chemdraw).

**Figure 3:**
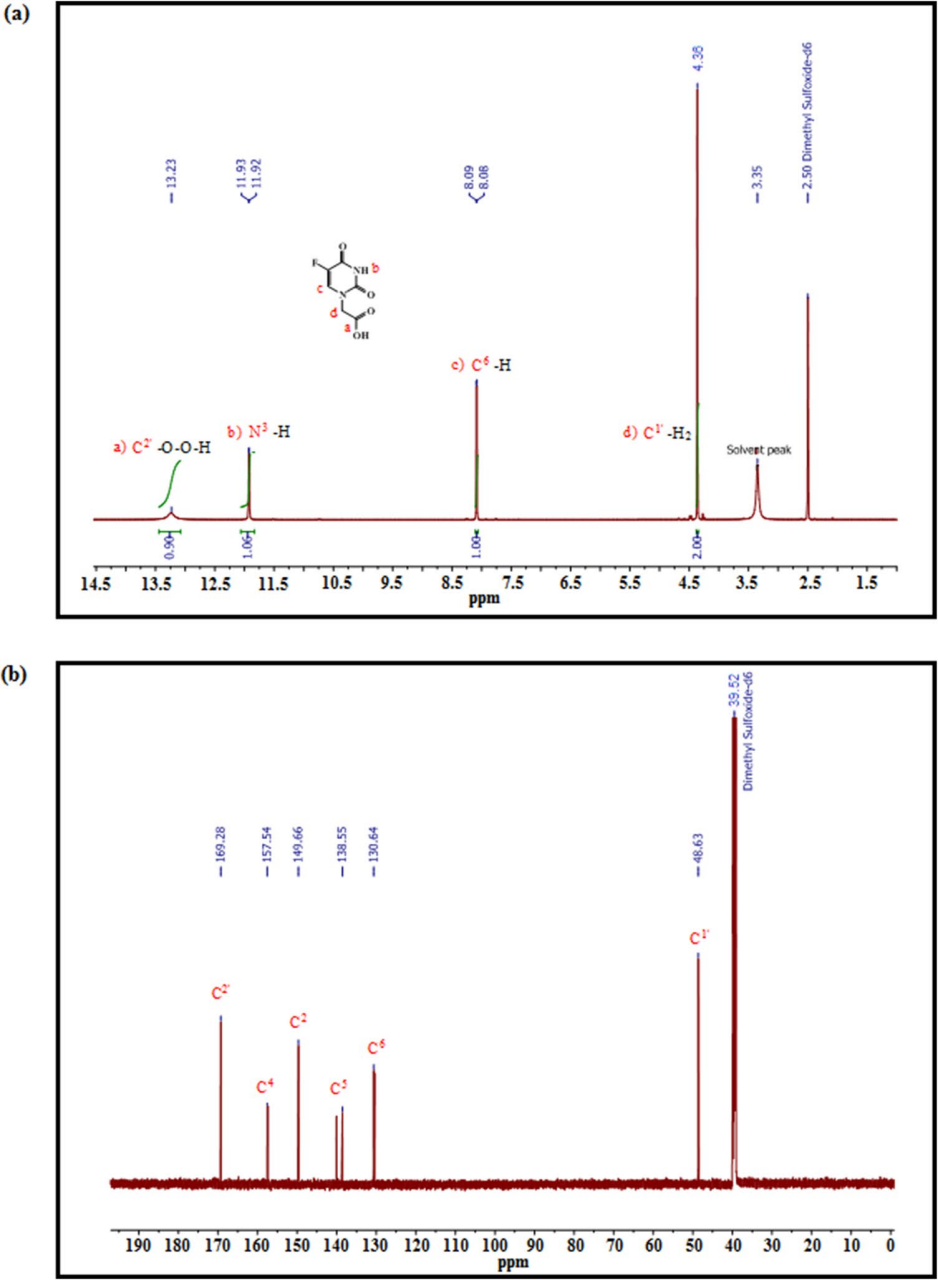
^1^H-NMR and ^13^C-NMR spectra of 5-FA. (**a**) ^1^H-NMR spectrum of 5-FA (600 MHz, solvent DMSO). The horizontal axis is the chemical shifts of proton signals in part per million (ppm) and the numbers above the axis reflect the abundance of the individual protons. ^1^H NMR (600 MHz, DMSO) δ 13.23 (s, 1H), 11.92 (d, *J* = 4.8 Hz, 1H), 8.08 (d, *J* = 6.7 Hz, 1H), 4.36 (s, 2H). (**b**) ^13^C-NMR spectrum of 5-FA (151 M Hz, solvent DMSO). The horizontal axis represents the energy or frequency of the radio waves absorbed by ^13^C atoms. ^13^C NMR (151 MHz, DMSO) δ 169.28 (s), 157.54 (s), 149.66 (s), 138.55 (s), 130.64 (s), 48.63 (s).

**Figure 4:**
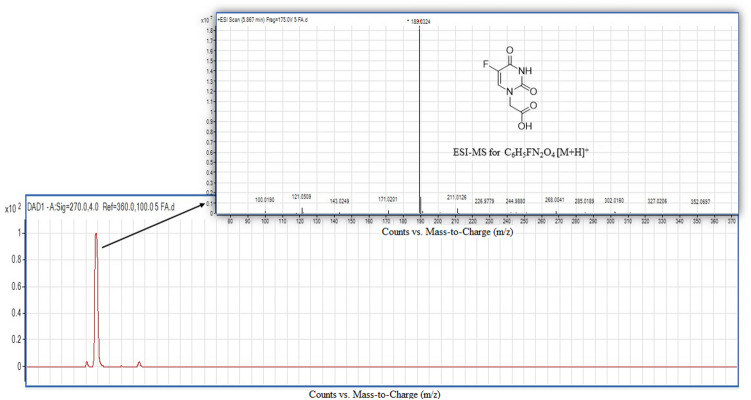
Mass spectrometric analysis of 5-FA. The molecular mass of 5-FA as determined by ESI-HRMS measurement was 189.0324 (m/z, protonated ion). The calculated molecular mass is 189.0306 [C_6_H_5_FN_2_O_4_; (M+H)^+^].

The conversion of 5-FU to 5-FA was performed as described in Sun et al.^37^. 5-FU reacted with α-chloroacetic acid in the presence of potassium hydroxide (KOH) and water at 60 °C (Scheme 2). The 5 h reaction yielded 83% of 5-FA. Nuclear magnetic resonance (NMR) spectroscopy and electrospray ionization-high resolution mass spectrometry (ESI-HRMS) were used to characterize the 5-FA. The substituted elements and the chain carbon atoms are highlighted and numbered according to their positions in the 5-FA molecule to ease the analysis of NMR spectroscopy spectra. According to the ^1^H-NMR spectrum (Fig. 3a), there is a peak with δ_H_ at 13.23 ppm, which corresponds to a carboxyl group in C2′. The presence of two oxygen atoms at C2′ causes deshielding of the hydrogen atom, and consequently moving the signal to the downfield. The area under the curve (AUC) of the peak is approximately 1, showing that the peak was generated by one hydrogen atom. The doublet with δ_H_ at 11.93 and 11.92 ppm seems to correspond to the hydrogen atom bound to N^3^, considering that it was created by only one hydrogen atom based on the AUC of the peak. This hydrogen atom is under influence of strong electronegative atoms in close proximity with it, which are the two oxygen atoms linked to C^2^ and C^4^, and caused the downfield position of the hydrogen atom. Additionally, there is no other hydrogen atom in the neighbourhood of this hydrogen atom according to the N + 1 rule. Another doublet with δ_H_ at 8.09 and 8.08 ppm also corresponds to one single hydrogen atom as predicted from the AUC. The chemical shift to downfield suggests the proximity to an electronegative atom. By comparing the J_3_ coupling constant (6.7 Hz) with J_4_ from the previous doublet, it can be concluded that this is larger, thus indicating a hydrogen or fluorine atom in position J_3_. The peak with a signal of δ_H_ at 4.36 ppm, and AUC of 2 indicates that the signal corresponds to two hydrogen atoms. Considering the chemical shift displacement, it is reasonable to conclude that there is a weak electron withdrawer neighbour, or it suffers the influence of both strong electron-withdrawing and strong electron-donating neighbours. This peak was then assigned to hydrogen atom in C1′ since it is influenced by both the electron-donor N^1^ and C^2′^ (Fig. 3a). The hydroxyl peak with δ_H_ at 13.23 ppm corresponds to the carboxyl group of 5-FA. Additionally, the signals of δ_H_ at 11.92 and 8.08 ppm, respectively, refer to the NH and CH protons in the 5-FA ring. These data are consistent with the number of atom carbon resonances observed in the ^13^C-NMR spectrum (Fig. 3b). In ^13^C-NMR spectrum, C^5^ represents a doublet. The fluorine atom connected to C5 is strongly electronegative. This electronegativity in the proximity of a carbon atom is in the origin of the double peak at 138.55 ppm and the peak in front. This doublet was assigned to C5 because this is a typical signal for F–C coupling, obeying the N + 1 rule and has a large coupling constant. Moreover, the molecular mass of 5-FA determined by ESI-HRMS was 189.0324 Da (m/z), which is in high agreement with the calculated molecular mass 189.0306 Da (C_6_H_5_FN_2_O_4_ [M + H]^+^), indicating successful conversion of 5-FU to 5-FA (Fig. 4).

### Conjugation of 5-FA to tHBcAg VLNP and CPP-tHBcAg VLNP

**Figure 5:**
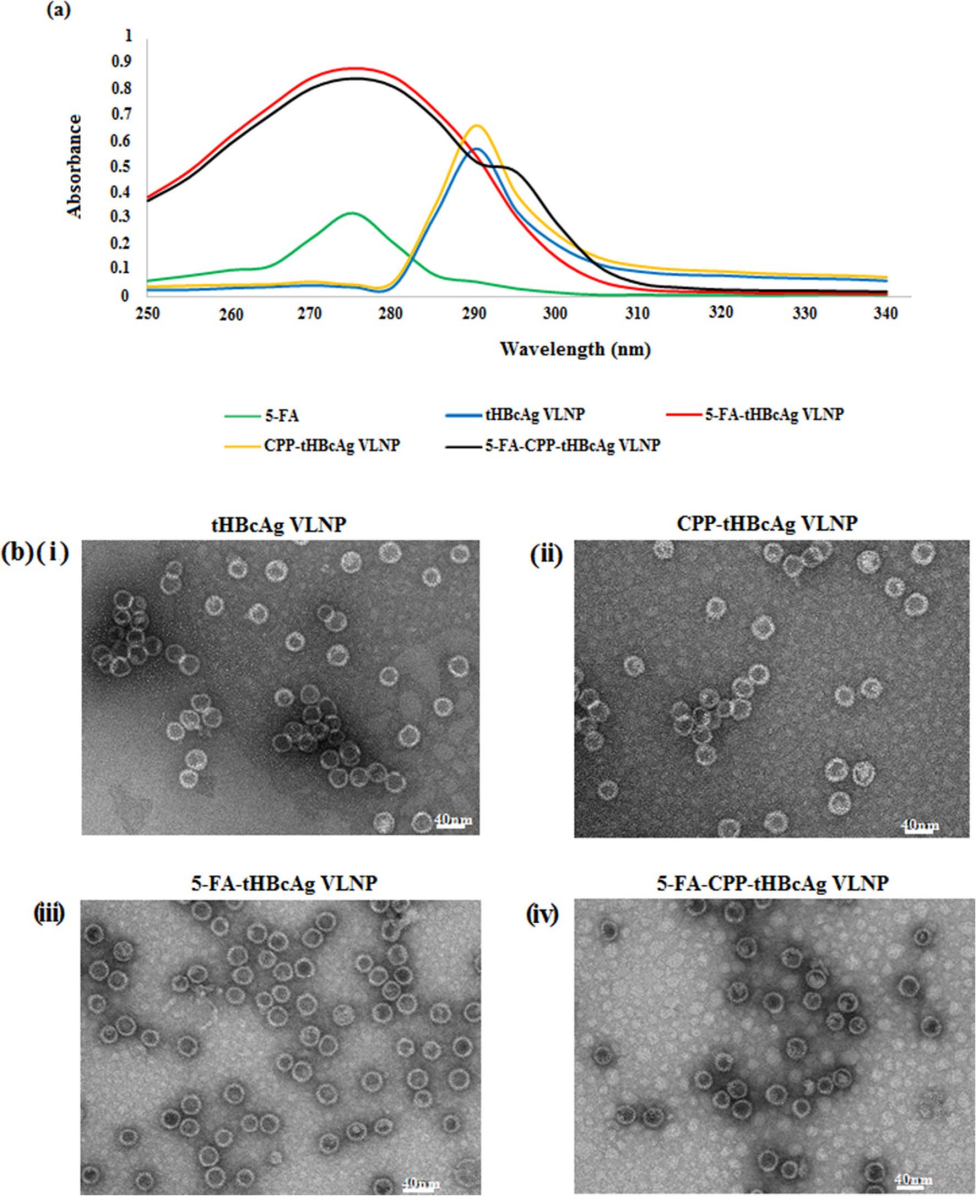
Conjugation of 5-FA and CPP onto tHBcAg VLNPs. CPP (NRPDSAQFWLHH) was co-synthesized with the nanoglue (SLLGRMKGA), and these two sequences were separated by a GGG-linker to produce a 24-residue peptide (NRPDSAQFWLHHGGGSLLGRMKGA). In the presence of the zero-length cross-linker EDC and sulfo-NHS, the nanoglue binds specifically at the tip of tHBcAg dimer, and the primary amine group of the Lys residue of the nanoglue was cross-linked to the adjacent carboxyl group from either Asp or Glu residues located at the tip of tHBcAg dimer (Scheme 1). (**a**) UV–visible spectra of 5-FA, tHBcAg VLNP, CPP-tHBcAg VLNP, 5-FA-tHBcAg VLNP and 5-FA-CPP-tHBcAg VLNP. (**b**) Electron micrographs of tHBcAg VLNP. Nanoparticles formed by (i) tHBcAg VLNP, (ii) CPP-tHBcAg VLNP, (iii) 5-FA-tHBcAg VLNP, and (iv) 5-FA-CPP-tHBcAg VLNP were stained with uranyl acetate and observed under a transmission electron microscope. White bars indicate 40 nm.

The conjugation of 5-FA to tHBcAg VLNP and CPP-tHBcAg VLNP was performed via a two-step carbodiimide method using EDC and Sulfo-NHS. The carboxyl group of 5-FA reacted with Sulfo-NHS in the presence of carbodiimide EDC to form a semi-stable Sulfo-NHS ester. This intermediate was then linked covalently to the primary amine (NH_2_) group exposed on the surface of tHBcAg VLNP and CPP-tHBcAg VLNP, to form an amide bond. The nanoparticles conjugated with 5-FA, namely 5-FA-tHBcAg VLNP and 5-FA-CPP-tHBcAg VLNP, were purified using sucrose density gradient ultracentrifugation, and the absorbance at 275 nm (A_275_) was measured spectrophotometrically. In comparison to unconjugated tHBcAg VLNP, 5-FA-tHBcAg VLNP and 5-FA-CPP-tHBcAg VLNP showed a remarkably higher absorbance at 275 nm, indicating that the nanoparticles were successfully conjugated with 5-FA (Fig. 5a). The conjugation efficiency of 5-FA (CE_5FA_) was 3.86 ± 0.43%, amounting to approximately 833 5-FA molecules per tHBcAg VLNP. Transmission electron microscopic analysis of the 5-FA-tHBcAg VLNP and 5-FA-CPP-tHBcAg VLNP revealed that the nanoparticles were intact with icosahedral shape, demonstrating that they were stable throughout the 5-FA conjugation process (Fig. 5b).

### Cytotoxicity of 5-FA and various 5-FA formulations

**Figure 6:**
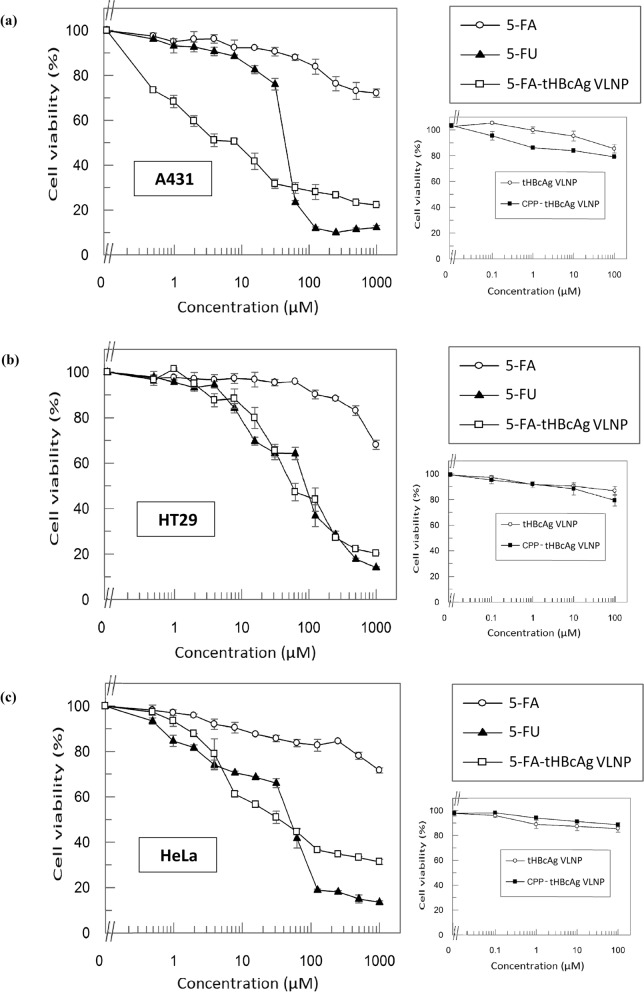
Cytotoxicity of various 5-FA formulations as determined by MTT assay. Viability of (**a**) A431 (**b**) HT29, and (**c**) HeLa cells after being treated with 5-FU, 5-FA and 5-FA-tHBcAg VLNP. 5-FA was less cytotoxic to all the tested cell lines as compared to the reference drug, 5-FU. The cytotoxicity of 5-FA was significantly enhanced after conjugation to tHBcAg VLNP. Small graphs on the right show that tHBcAg VLNP and CPP-tHBcAg VLNP were not toxic to the tested cells. Data are expressed as mean ± SD of triplicate measurements.

**Figure 7:**
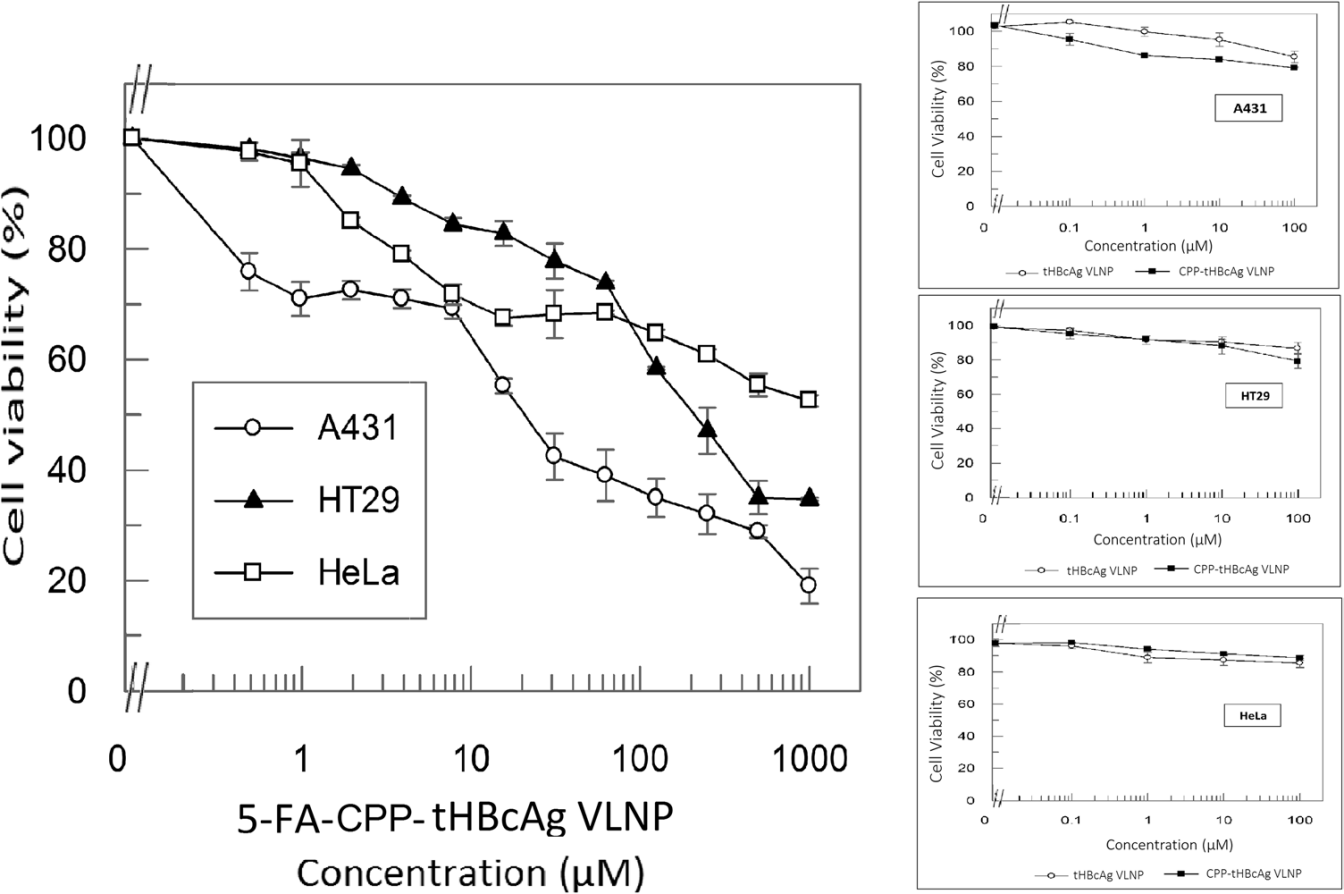
Selective cytotoxicity of 5-FA-CPP-tHBcAg VLNP on A431, HT29 and Hela cells. Viability of A431, HT29 and HeLa cells treated with 5-FA-CPP-tHBcAg VLNP. After the conjugation of CPP (NRPDSAQFWLHH) to the 5-FA-tHBcAg VLNP, the cytotoxic effect of 5-FA-CPP-tHBcAg VLNP became selective, in which it was more cytotoxic to A431 cells as compared to HT29 and HeLa cells. The cytotoxic effect of 5-FA-CPP-tHBcAg VLNP in HT29 was higher compared to HeLa cells. Small graphs on the right show that tHBcAg VLNP and CPP-tHBcAg VLNP were not toxic to the tested cells. Data are expressed as mean ± SD of triplicate measurements.

3-(4,5-dimethylthiazol-2-yl)-2,5,-diphenyltetrazoliumbromide (MTT) assay was performed to evaluate the cytotoxicity of 5-FA, 5-FA-tHBcAg VLNP and 5-FA-CPP-tHBcAg VLNP towards A431, HT29 and HeLa cells. 5-FU served as a positive control, and it had IC_50_ values of 47.02 ± 0.65 µM, 85.37 ± 1.81 µM and 43.34 ± 2.77 µM for A431, HT29 and HeLa cells, respectively (Fig. 6). The IC_50_ value of 5-FA in all the three cell lines could not be determined even though its concentration was increased to 1 mM, indicating that the 5-FA was significantly less toxic compared to 5-FU. After the conjugation of 5-FA to the nanoparticles, the IC_50_ values of 5-FA-tHBcAg VLNP in A431, HT29 and HeLa cells were 3.26 ± 0.29 µM, 71.96 ± 1.97 µM and 36.94 ± 2.52 µM, respectively. This shows that 5-FA-tHBcAg VLNP is more toxic than 5-FU, and the cytotoxicity of these two compounds is not in EGFR-dependent manner as their inhibitory activities on HeLa cells (containing the least EGFR) are higher than those on HT29 cells (containing the intermediate level of EGFR). Interestingly, when CPP was conjugated to 5-FA-tHBcAg VLNP, the resulting formulation, 5-FA-CPP-tHBcAg VLNP, demonstrated EGFR-dependent inhibitory activities, although its toxicity is lower than the former. A431 cells expressing the highest number of EGFR had the lowest IC_50_ value, 21.98 ± 3.8 µM, followed by HT29 cells which showed an IC_50_ value of 215.16 ± 1.89 µM. The IC_50_ value of 5-FA-CPP-tHBcAg VLNP could not be determined up to 1 mM in HeLa cells, which contain the lowest level of EGFR among the three cell lines (Fig. 7). The negative controls, tHBcAg VLNP and CPP-tHBcAg VLNP, were not toxic to the three tested cell lines.

### Apoptotic activity of 5-FA formulations on A431 cells

**Figure 8:**
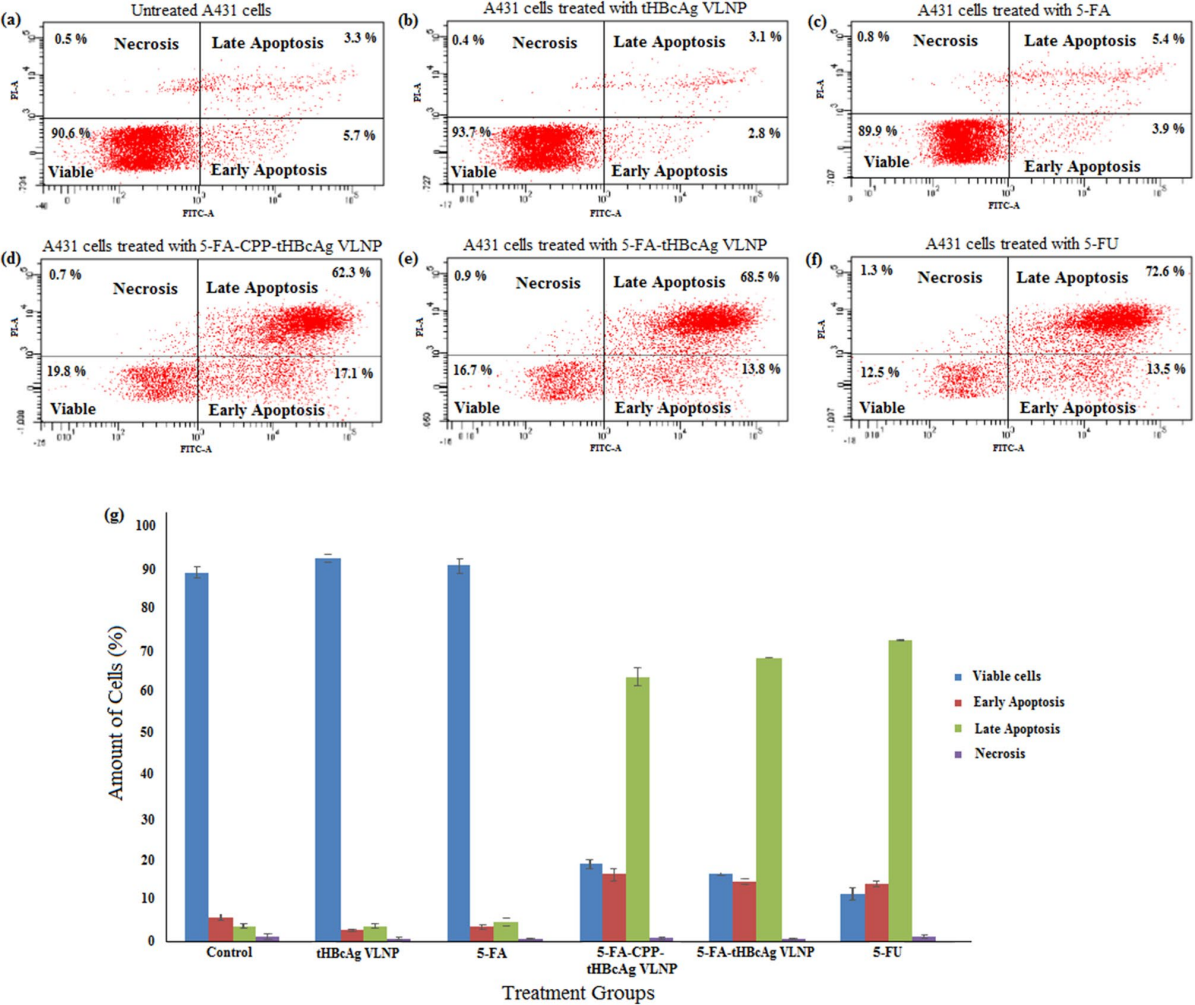
Flow cytometry analysis of apoptosis in A431 cells treated with various 5-FA formulations. A431 cells were treated with various 5-FA formulations and the reference drug (5 FU). After staining with FITC-Annexin V and PI, the cells were analyzed using a flow cytometer. The dot plots are the (**a**) untreated A431 cells which served as a control group, (**b**) A431 cells treated with tHBcAg VLNP, (**c**) A431 cells treated with 5-FA, (**d**) A431 cells treated with 5-FA-CPP-tHBcAg VLNP, (**e**) A431 cells treated with 5-FA-tHBcAg VLNP and (**f**) A431 cells treated with 5-FU. (**g**) Percentage of viable, early apoptotic, late apoptotic and necrotic cells treated with various 5-FA formulations and 5-FU. The results are shown as mean ± SD of three independent experiments.

Flow cytometry was performed to study the induction of A431 cell apoptosis by various 5-FA formulations. FITC annexin V staining was used as a marker for apoptotic cell death, and 5-FU served as the reference drug. The results showed that very few cells were apoptotic in untreated cells, as well as cells treated with tHBcAg VLNP and 5-FA (Fig. 8). The cells treated with 5-FU, which served as a positive control, underwent apoptosis. The percentage of early apoptosis increased significantly in the cells treated with 5-FA-tHBcAg VLNP, 5-FA-CPP-tHBcAg VLNP and 5-FU (Fig. 8g). Furthermore, the percentage of cells at late apoptotic stage increased significantly to 68.5%, 62.3%, and 72.6%, in the cells treated with 5-FA-tHBcAg VLNP, 5-FA-CPP-tHBcAg VLNP and 5-FU, respectively. Overall, these data demonstrated that 5-FA after being conjugated to tHBcAg VLNP has an anti-proliferative effect on A431 cells by inducing apoptosis.

## Discussion

Chemotherapy is one of the most widely used methods for cancer treatments. However, low specificities of chemotherapeutic agents towards cancer cells in the human body always lead to poor cancer prognosis and mortality^38,39^. Therefore, specific delivery of chemotherapeutic drugs to cancer cells and tissues with minimal side effects on healthy tissues is a promising field for cancer treatments. EGFR is expressed abundantly in a broad spectrum of human cancers such as ovary, breast, lung, bladder, colon and skin cancers^40–42^. Hence, ligands that interact specifically with EGFR have gained popularity. Previously, we have isolated a CPP with the amino acid sequence NRPDSAQFWLHH from a phage displayed peptide library via biopanning^13^. The internalization of the CPP into A431 cells was inhibited by the anti-EGFR antibody^13^. Fluorescence activated cell sorting (FACS) analysis on EGFR performed by Beusechem et al.^36^ revealed that A431 cells expressed the highest level of EGFR with a relative median fluorescence (RMF) value of 13.5, followed by HT29 cells (RMF: 7.3) and HeLa cells (RMF: 3.5). This is in good agreement with our study using immunofluorescence microscopy, which demonstrated that the EGFR expression level was the highest in A431, followed by HT29 and HeLa cells. In the present study, we have further demonstrated that the degree of CPP internalization is proportional to the amount of EGFR expressed on different cancer cells, with A431 showing the highest fluorescence intensity, followed by HT29 and HeLa cells. This further justifies that the internalization of the CPP into these cell lines is EGFR-dependent.

With an aim to enhance the therapeutic efficacy while minimizing undesired side effects of chemotherapy, researchers have designed various types of nanoparticles for incorporation of anticancer drugs, and specific drug delivery. VLNPs have attained much interest in the smart drug delivery system. The advantages of VLNPs over synthetic nanomaterials are their stable and highly ordered structural architecture in nanosize scale, which enhance tumor permeability and retention^43^, monodispersity and ease of production^44^, and well-defined interfaces for functionalization. Overall, VLNPs are less toxic, more stable, and more uniform as compared to non-viral based nanoparticles such as metal nanoparticles, liposomes and polymer particles^20,45,46^. To demonstrate the efficiency of the CPP as a targeting moiety to deliver VLNPs into cells expressing different levels of EGFR, the CPP was co-synthesized with the nanoglue, and covalently linked to tHBcAg VLNP. Immuno-fluorescence microscopy demonstrated that tHBcAg VLNP displaying the CPP successfully delivered the nanoparticle into A431, HT29 and HeLa cells in EGFR-dependent manner, which paves the way for the application of the CPP to deliver VLNPs to cancer cells overexpressing EGFR.

As one of the most commonly prescribed anti-cancer agents, 5-FU, either alone or in combination with other anticancer agents, has been commonly applied for the treatments of various types of cancers^47^. A variety of chemically modified 5-FU derivatives have been synthesized to enhance their antitumor efficacy^48,49^, but their irregular oral absorption, low bioavailability and lack of specificity often result in poor clinical therapeutic outcomes^5–7^. In order to improve the oral bioavailability, a wide variety of polymers have been synthesized for direct or indirect attachment of 5-FU and its derivatives^50,51^. In the present study, 5-FA was introduced as a 5-FU derivative as it has been reported to be highly effective and less toxic^52^. To synthesize 5-FA, 5-FU was reacted with chloroacetic acid in the presence of KOH. NMR and ESI-HRMS analyses confirmed the successful conversion of 5-FU to 5-FA. In order to deliver the 5-FA to cancer cells overexpressing EGFR, it was then conjugated to tHBcAg VLNP displaying the CPP. The conjugation was confirmed with spectrophotometer, and TEM analysis revealed that conjugation of 5-FA and CPP to tHBcAg VLNP has no undesired effect on the icosahedral structure of the nanoparticle.

In the present study, the cytotoxic effects of various 5-FA formulations on cells expressing different levels of EGFR were evaluated by MTT assay. The results clearly showed that the free 5-FA is less toxic than the reference drug, 5-FU. A study by Imoto et al.^53^ revealed that 5-FU entered Caco-2 cells by passive diffusion. The terminal carboxyl group in 5-FA renders the molecule charged at physiological conditions, slightly more hydrophilic and higher polarity than 5-FU^54^. As a result, 5-FA is unable to diffuse easily through the lipid core of the plasma membrane^55,56^. These properties reduce 5-FA’s cytotoxicity towards the cells. Moreover, the methyl-carboxyl group at N1 in the uracil group changes the 5-FU molecule in a way that makes it no longer a direct pyrimidine analog^57,58^. However, the cytotoxic effect of 5-FA was enhannced after being conjugated to tHBcAg VLNP, due to the non-specific binding and uptake of tHBcAg VLNP into the cells^59^, and subsequently the nanoparticle was hydrolyzed to release active 5-FU in the cells^60,61^. The non-specific distribution of 5-FA-tHBcAg VLNP in all the three cancer cell lines has become a drawback for this formulation, although the cytotoxic effect of 5-FA increased significantly upon conjugation onto tHBcAg VLNP. To address this problem, the CPP that targets EGFR was conjugated onto the 5-FA-tHBcAg VLNP. The resulting conjugate, namely 5-FA-CPP-tHBcAg VLNP displayed a much higher cytotoxicity in A431 cells compared to HT29 cells, followed by a very low cytotoxicity in HeLa cells. This EGFR-dependent manner was also observed for CPP-tHBcAg VLNP in the three cell lines (Fig. 2). The enhanced cytotoxicity of 5-FA-CPP-tHBcAg VLNP is believed to be caused by a specific interaction between the CPP displayed on the VLNP with EGFR.

5-FU induces apoptosis in cancer cells^62–64^, and 5-FA is believed to kill tumor cells with a similar mechanism as 5-FU^65^. Therefore, to confirm the apoptotic activity induced by various 5-FA formulations, FITC Annexin V/PI (propidium iodide) flow cytometry assay was performed on A431 cells to identify externalization of phosphotidylserine (PS), a prevalent sign of apoptosis. FITC Annexin V is designed as a probe with high affinity for PS, for the detection of early apoptosis^66^. The results showed that the percentage of total apoptotic cells was far more than necrotic cells in A431 cells treated with the 5-FA formulations. This indicates that the 5-FA formulations markedly induced apoptosis, but to a much lesser extent, to cause necrotic cell death. Therefore, together with the targeting property of CPP, 5-FA-CPP-tHBcAg VLNP could be used as a therapeutic agent that induces apoptosis in cancer cells overexpressing EGFR.

In summary, 5-FA was successfully synthesized from 5-FU, and the former was significantly less toxic than the latter in A431, HT29 and HeLa cells. However, the cytotoxicity of 5-FA was greatly enhanced upon conjugation to tHBcAg VLNP. Besides, the specificity of tHBcAg VLNP increased significantly by conjugating the CPP that targets EGFR on the surface of the nanoparticle via the nanoglue concept. The resultant 5-FA-CPP-tHBcAg VLNP internalized cell lines expressing EGFR in relation to the amount of the receptor. Overall, tHBcAg VLNP together with the EGFR-CPP is favorable for targeted drug delivery system. Apart from the CPP and 5-FA, other tumor targeting moieties and chemotherapeutic drugs can conveniently be presented on the outer surface of tHBcAg VLNP using appropriate cross-linkers, expanding the variety of anti-cancer drugs to be delivered specifically to different types of cancer cells.

## Materials and methods

### Cell culture

The A431 (human squamous carcinoma), HT29 (human colorectal cancer) and HeLa (cervical cancer) cells were cultured in Dulbecco’s Modified Eagle’s medium (DMEM) containing 10% (v/v) fetal bovine serum (FBS) (Sigma Aldrich, St. Louis, Missouri, USA). All cells were cultured at 37 °C in an incubator with humidified atmosphere and 5% CO_2_.

### Analysis of EGFR expression using immunofluorescence microscopy

A431, HT29 and HeLa cells (2 × 10^5^ cells/well) were grown in six-well plates containing sterile glass coverslips, and incubated at 37 °C for 24 h. Subsequently, the medium was aspirated and the cells were washed three times with PBS (2.7 mM KCl, 137 mM NaCl, 8.1 mM Na_2_HPO_4_, 1.47 mM KH_2_PO_4_; pH 7.4). Then, the cells were fixed with 3.7% (w/v) paraformaldehyde in PBS at room temperature (RT) for 10 min. After three washes, the cells were blocked with BSA (0.2 mg/mL, Amresco, Ohio, USA) in PBS at RT for 1 h. Subsequently, the cells were incubated with rabbit anti-EGFR monoclonal antibody (10 µg/mL, Cetuximab clone C225; Merck, Billerica, Massachusetts, USA) in PBS containing BSA (0.2 mg/mL) for 2 h at RT. After three washes, the cells were incubated with the goat anti-rabbit IgG conjugated to FITC [1:50 dilutions in PBS containing BSA (0.2 mg/mL); Novus Biologicals, Littleton, Colorado, USA] for 1 h at RT. After that, the cells were washed and stained with Hoechst 33342 prior to viewing under a fluorescence microscope.

### Selective internalization property of CPP (NRPDSAQFWLHH) in cell lines expressing different levels of epithelial growth factor receptor (EGFR)

A431, HT29 and HeLa cells (2 × 10^5^ cells/well) were grown in six-well plates containing sterile glass coverslips and incubated at 37 °C for 24 h. Subsequently, the medium was aspirated, and 0.25 mM FITC-NRPDSAQFWLHH (Mimotopes Pty Ltd, Clayton, Victoria, Australia) was added to the cell lines accordingly, prior to a 16 h-incubation at 37 °C in a CO_2_ incubator. The cells were then rinsed thoroughly with PBS prior to 10 min-fixation with 3.7% (w/v) paraformaldehyde in PBS, followed by staining of the cell nuclei with Hoechst 33342 (Ex_360nm_ and Em_460nm_; 2 drops/mL in PBS; Life Technologies, Carlsbad, California, USA) for 10 min at 25 °C. Then, the cells were thoroughly rinsed with PBS. Cover slips were placed onto the glass slides containing a drop of mounting medium [0.1 M propyl gallate, 20 mM Tris–HCl, 90% (v/v) glycerol; pH 8.5], and sealed with nail polish. The cells were then observed under a fluorescence microscope (Olympus X5, Olympus, Tokyo, Japan).

### Conjugation of peptide NRPDSAQFWLHHGGGSLLGRMKGA to tHBcAg VLNP, and cellular uptake of CPP-tHBcAg VLNP into A431, HT29 and HeLa cell lines

tHBcAg VLNP was produced and purified as described in Tan et al.^32^ and Yoon et al.^67^. Peptide NRPDSAQFWLHHGGGSLLGRMKGA was conjugated at the spike of the tHBcAg VLNP by mixing tHBcAg: peptide (1:1) in phosphate buffer (25 mM NaH_2_PO_4_/Na_2_HPO_4_, pH 7) in the presence of EDC and Sulfo-NHS as described in Gan et al.^13^. The conjugated product was dialyzed against phosphate buffer (pH 7) to remove the excessive cross-linkers, and concentrated with VIVASPIN 6 (30 kDa cut-off polyethersulfone membrane; VIVASCIENCE, Germany) at 4500×*g*, 4 °C. The CPP-tHBcAg VLNP (250 µg/mL) was applied to A431, HT29 and HeLa cells in order to study its rate of internalization into these cells. The cells were incubated at 37 °C for 16 h, washed, fixed, and permeabilized with ice cold methanol at − 20 °C for 6 min. Then, the cells were incubated with the mouse anti-HBcAg monoclonal antibody [1:100 dilutions in PBS containing BSA (0.2 mg/mL); Santa Cruz Biotechnology, Dallas, Texas, USA] for 1 h at RT, followed by incubation with FITC-conjugated goat anti-mouse antibody (1: 100 dilutions in PBS containing 0.2 mg/mL BSA; BD Biosciences, San Jose, CA, USA) for another 1 h at RT. After that, the cells were washed and stained with Hoechst 33342 prior to viewing under a fluorescence microscope.

### Synthesis and characterization of 5-fluorouracil-1-acetic acid (5-FA)

The preparation of 5-FA was performed as described in Sun et al.^37^ with slight modifications. 5-FU (1.6 g, 12.3 mmol; Nacalai Tesque, Kyoto, Kyoto Prefacture, Japan) was added into a 50 mL-round-bottom flask containing 8 mL aqueous KOH solution (2.5 g, 44.6 mmol), stirred and heated at 80 °C for 30 min, before α-chloroacetic acid (1.2 g, 12.7 mmol; Sigma Aldrich, St. Louis, Missouri, USA) was added drop by drop into the reaction. The reaction mixture was stirred and heated at 60 °C for 5 h. The progress of the reaction was monitored using thin layer chromatography (TLC) (Supplementary Fig. S2). The cooled reaction mixture was then adjusted to pH 5.5 with 2 M HCl solution. The precipitate formed was removed, and the solution was then adjusted to pH 2.0 and kept at 4 °C for 18 h. The precipitate was collected as a crude product, and recrystallized with water to produce 5-FA as a white crystalline product.

### Conjugation of 5-FA to tHBcAg VLNP and CPP-tHBcAg VLNP

The conjugation of 5-FA to CPP-tHBcAg VLNP was performed as described in Biabanikhankahdani et al.^34^ with some modifications. The carboxyl group of 5-FA was activated by dissolving 5-FA (5 mg), sulfo-NHS (20 mg) and EDC (20 mg) in sodium phosphate buffer (25 mM NaH_2_PO_4_/Na_2_HPO_4_, pH 6.0; 5 mL) at RT for 8 h. After that, the pH of the solution was increased to 7.4 with NaOH, and the CPP-tHBcAg VLNP (3 mg) in sodium phosphate buffer was added. The mixture was then incubated with gently agitation at 4 °C overnight, followed by sucrose density gradient ultracentrifugation (8–40%; 210,000×*g*, for 5 h at 4 °C) as described in Tan et al.^32^. Simultaneously, activated 5-FA was also added to tHBcAg VLNP, and the same conjugation procedure was applied. UV–visible measurements of tHBcAg VLNP, 5-FA, 5-FA-tHBcAg VLNP, 5-FA-CPP-tHBcAg VLNP and CPP-tHBcAg VLNP were determined using a spectrophotometer (Jenway 7315, Staffordshire, UK). The absorbance at wavelength 275 nm, which corresponds to the amount of 5-FU was measured at RT. The conjugation efficiency of 5-FA (CE_5-FA_) and the number of 5-FA (N_5-FA_) molecules conjugated to the nanoparticle were calculated using Eqs. (1) and (2), respectively.

1 $${\text{CE}}_{{{5} - {\text{FA}}}} \% = \left( {{\text{Weight}}_{{{5} - {\text{FA}}}} /{\text{ Weight}}_{{\text{tHBcAg VLNP}}} } \right) \, \times { 1}00\%$$

2 $${\text{N}}_{{\text{5} - \text{FA }}} = {\text{CE}}_{{\text{5} - \text{FA}}} \times \, \left( {{\text{Mw}}_{{{\text{tHBcAg}}\,\,{\text{particle}}}} /{\text{Mw}}_{{\text{5} - \text{FA}}} } \right)$$

M_w_ represents molecular weight.

### Transmission electron microscopy (TEM)

tHBcAg VLNP, 5-FA-tHBcAg VLNP, CPP-tHBcAg VLNP and 5-FA-CPP-tHBcAg VLNP (0.35 mg/mL; 15 µL) were absorbed onto 200-mesh carbon coated copper grids. Negative staining of the particles was done using freshly prepared and filtered uranyl acetate solution [2% (w/v) in distilled water; 15 µL] for 5 min, prior to the viewing of grids under a TEM (Hitachi H-7700, Japan).

### Cytotoxicity of 5-FA formulations

Cytotoxicity of free 5-FU, 5-FA, 5-FA-tHBcAg VLNP, and 5-FA-CPP-tHBcAg VLNP was determined using the cell viability MTT assay. A431, HT29 and HeLa cells (2.0 × 10^4^ cells/well) were seeded in 96-well plates, and incubated for 24 h. Subsequently, the culture media were discarded, and the cells were added with media (100 µL) containing free 5-FU, 5-FA, 5-FA-tHBcAg VLNP and 5-FA-CPP-tHBcAg VLNP at twofold serial dilution (0.049 µM-1000 µM), and incubated for 72 h. After the incubation, MTT reagent (5 mg/mL; 20 µL) prepared in PBS was pipetted into the wells and incubated for 3 h. Dimethyl sulfoxide (DMSO; 100 µL) was then added to the wells and incubated for 15 min to dissolve the formazan crystal in the viable cells. A_570 nm_ was measured using a microtiter plate reader (Elx800, Bio-Tek Instruments, USA). The cytotoxicity of tHBcAg VLNP and CPP-tHBcAg VLNP was studied as negative controls.

### Determination of apoptotic activity in A431 cells induced by 5-FA formulations

A431 cells (2.0 × 10^5^ cells/well) were seeded in 6-well plates, and incubated for 24 h. The culture media were then discarded, and the cells were added with media (2 mL) containing free 5-FU, 5-FA, 5-FA-tHBcAg VLNP and 5-FA-CPP-tHBcAg VLNP at equivalent concentration of 5-FU and 5-FA (125 µM), and incubated for 48 h. After incubation, apoptotic activity of the cells was determined using FITC Annexin V Apoptosis Detection Kit (BD Biosciences, San Jose, California, USA). FITC Annexin V staining was performed on the cells according to the manufacturer’s protocol, and the fluorescence signal was examined using a flow cytometer (BD FACS-CantoIITM, Becton Dickinson, USA). The data were analyzed using the FACS Diva software Version 6.1.3.

### Statistical analysis

Statistical analysis was performed using the SPSS program. Values of p < 0.01 are considered statistically significant.

## Supplementary information

Supplementary information
