# Supplementary material for: Targeted delivery of 5-fluorouracil-1-acetic acid (5-FA) to cancer cells overexpressing epithelial growth factor receptor (EGFR) using virus-like nanoparticles
Source: Sci Rep. 2020 Oct 8;10:16867. doi: 10.1038/s41598-020-73967-4 (PMC7545207; doi:10.1038/s41598-020-73967-4)
Supplement: Supplementary file 1 — Supplementary information [file 41598_2020_73967_MOESM1_ESM.pdf]

Targeted Delivery of 5-Fluorouracil-1-Acetic Acid (5-FA) to Cancer Cells Overexpressing Epithelial Growth Factor Receptor (EGFR) using Virus-Like Nanoparticles

Bee Koon Gan[1], Kamal Rullah[2], Chean Yeah Yong[1], Kok Lian Ho[3], Abdul Rahman Omar[1,4], Noorjahan Banu Alitheen[1,5] & Wen Siang Tan[1,6*]

[1]Institute of Bioscience, Universiti Putra Malaysia, 43400 UPM Serdang, Selangor, Malaysia.

[2]Department of Pharmaceutical Chemistry, Kulliyyah of Pharmacy (KOP), International Islamic University Malaysia (IIUM), 25200 Kuantan, Pahang, Malaysia.

[3]Department of Pathology, Faculty of Medicine and Health Sciences, Universiti Putra Malaysia, 43400 UPM Serdang, Selangor, Malaysia.

[4]Department of Veterinary Pathology & Microbiology, Faculty of Veterinary Medicine, Universiti Putra Malaysia, 43400 UPM Serdang, Selangor, Malaysia.

[5]Department of Cell and Molecular Biology, Faculty of Biotechnology and Biomolecular Sciences, Universiti Putra Malaysia, 43400 UPM, Serdang Selangor, Malaysia.

[6]Department of Microbiology, Faculty of Biotechnology and Biomolecular Sciences, Universiti Putra Malaysia, 43400 UPM, Serdang Selangor, Malaysia.

*Correspondence and requests for materials should be addressed to W.S.T. (email: wstan@upm.edu.my)

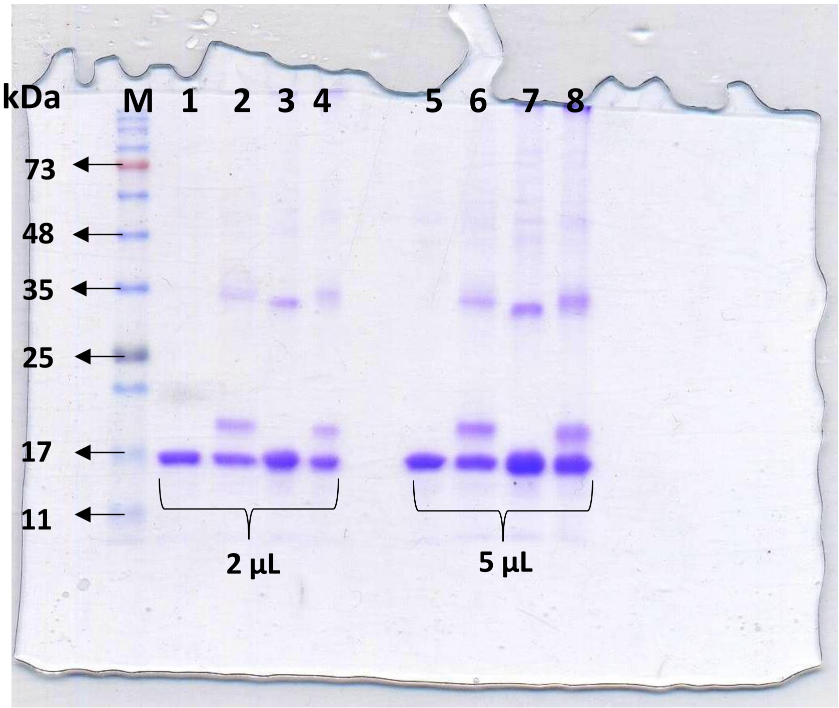

Figure S1. SDS-polyacrylamide gel of tHBcAg conjugated to CPP. The tHBcAg conjugated to CPP using EDC and sulfo-NHS was electrophoresed on 15% (w/v) SDS-polyacrylamide gel, and stained with CBB-R250. **Lanes M:** molecular mass markers (kDa), **1 & 5:** tHBcAg, **2, 4, 6 & 8:** tHBcAg plus CPP and cross-linker, **3 & 7:** tHBcAg plus cross-linker without CPP

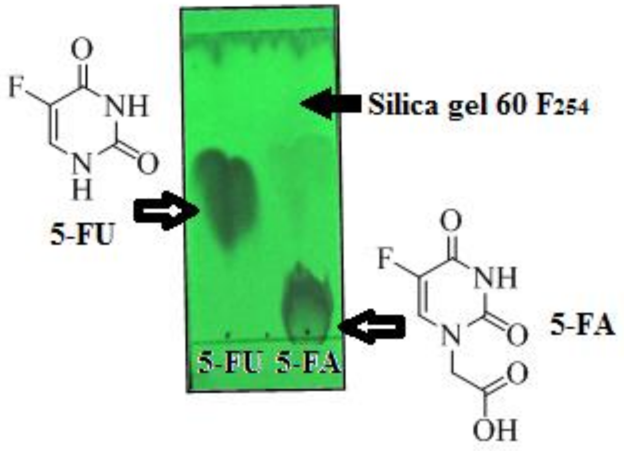

Figure S2. Thin layer chromatography (TLC) of 5-FU and 5-FA. The hydroxyl groups on the surface of silica gel make the stationary phase highly polar. 5-FA (carboxylic group of polar analyte) interacts strongly with the stationary phase, and moves slower compared to 5-FU (non-polar analyte), which interacts weakly with the stationary phase, and moves faster along with the solvent through the chromatography plate.
